# Analysis of codon usage patterns in open reading frame 4 of hepatitis E viruses

**DOI:** 10.1186/s43088-022-00244-w

**Published:** 2022-05-10

**Authors:** Zoya Shafat, Anwar Ahmed, Mohammad K. Parvez, Shama Parveen

**Affiliations:** 1 Centre for Interdisciplinary Research in Basic Sciences, Jamia Millia Islamia, New Delhi, 110025 India; 2 Centre of Excellence in Biotechnology Research, College of Science, King Saud University, Riyadh, Saudi Arabia; 3 Department of Pharmacognosy, College of Pharmacy, King Saud University, Riyadh, Saudi Arabia

**Keywords:** Hepatitis E virus (HEV), Open reading frame 4 (ORF4), Nucleotide composition, Synonymous codon usage, Mutational pressure, Natural selection

## Abstract

**Background:**

Hepatitis E virus (HEV) is a member of the family *Hepeviridae* and causes acute HEV infections resulting in thousands of deaths worldwide. The zoonotic nature of HEV in addition to its tendency from human to human transmission has led scientists across the globe to work on its different aspects. HEV also accounts for about 30% mortality rates in case of pregnant women. The genome of HEV is organized into three open reading frames (ORFs): ORF1 ORF2 and ORF3. A reading frame encoded protein ORF4 has recently been discovered which is exclusive to GT 1 isolates of HEV. The ORF4 is suggested to play crucial role in pregnancy-associated pathology and enhanced replication. Though studies have documented the ORF4’s importance, the genetic features of ORF4 protein genes in terms of compositional patterns have not been elucidated. As codon usage performs critical role in establishment of the host–pathogen relationship, therefore, the present study reports the codon usage analysis (based on nucleotide sequences of HEV ORF4 available in the public database) in three hosts along with the factors influencing the codon usage patterns of the protein genes of ORF4 of HEV.

**Results:**

The nucleotide composition analysis indicated that ORF4 protein genes showed overrepresentation of C nucleotide and while A nucleotide was the least-represented, with random distribution of G and T(U) nucleotides. The relative synonymous codon usage (RSCU) analysis revealed biasness toward C/G-ended codons (over U/A) in all three natural HEV-hosts (human, rat and ferret). It was observed that all the ORF4 genes were richly endowed with GC content. Further, our results showed the occurrence of both coincidence and antagonistic codon usage patterns among HEV-hosts. The findings further emphasized that both mutational and selection forces influenced the codon usage patterns of ORF4 protein genes.

**Conclusions:**

To the best of our knowledge, this is first bioinformatics study evaluating codon usage patterns in HEV ORF4 protein genes. The findings from this study are expected to increase our understanding toward significant factors involved in evolutionary changes of ORF4.

**Supplementary Information:**

The online version contains supplementary material available at 10.1186/s43088-022-00244-w.

## Background

Hepatitis E virus (HEV) is a small RNA virus, belonging to the *Hepeviridae* family. Hepatitis E is potentially a serious acute disease caused by the agent HEV [1, 2]. HEV is primarily transmitted through contaminated water sources or through the consumption of infected or undercooked meat products derived from animals (swine, deer, or wild boar) [3, 4]. The HEV contains a positive-sense, single-stranded RNA molecule of approximately 7.2 kB in length, flanked by 5′ and 3′ untranslated regions (UTR) [5]. The genome possesses a 7-methylguanine cap at the 5′ end and a poly(A) tail at the 3′ end and encodes three open reading frames (ORFs), i.e., ORF1, ORF2 and ORF3. ORF1 encodes the largest non-structural polyprotein having multifunctional domains, required for viral replication [6, 7]. The reading frame ORF2 codes for the capsid protein [8]. The ORF3 encodes the phosphorylated protein having multiple functions [9, 10]. HEV genotype 1 (GT 1) isolates have been recently identified with an additional reading frame (ORF4), which encodes ORF4 protein only during ER stress [11]. This newly identified ORF4 is exclusive to HEV GT 1 [11]. ORF4 has been demonstrated to play a significant functional role in the replication cycle of GT 1 HEV. Evidence suggests that ORF4 interacts with multiple viral and host proteins to enhance virus replication [11, 12].

The present study analyzed the compositional biasness in terms of nucleotide composition and synonymous codon usage patterns of the HEV ORF4 protein genes. The prevalence of degeneracy in the genetic code allows more than one codon to encode for a specific amino acid. Thus, alternative codons encoding the same amino acid are termed as synonymous codons. Interestingly, in viruses, the preference of some codons over the others has been well documented. This phenomenon refers to codon usage bias (CUB) [13, 14]. CUB is considered as an important force in the evolution of viral genomes. Factors influencing the CUB include mutational pressure, natural selection, G + C content, secondary protein structure and selective transcription replication [15–18]. Previous reports have suggested that natural selection and directional mutation pressure are two major mechanisms that account for codon usage variation among viral genomes [15, 19–21]. However, mutational bias, rather than natural selection, found to be a dominant factor affecting the codon usage patterns in some RNA viruses [22–25]. The development of a disease is caused by the complex interaction among various factors, which includes pathogen’s virulence, host organism defense response and environmental aspects [26, 27]. These mentioned factors play role in addition to CUB decide the outcome of the host–pathogen interaction or relationship [28, 29]. The pathogens can better adapt to their hosts as well as its environment by allowing certain evolutionary changes which is reflected by their CUB patterns. Moreover, the efficiency of a pathogen to infect its host is significantly dependent on codon optimization process. This is because codon optimization affects the growth of a pathogen in its environment [28]. The similar codon usage pattern among virus and its hosts may overall influence the virus’s fitness, evasion from host’s immune system and evolution [30, 31]. Therefore, the study of codon usage in viruses can reveal important information about virus evolution, regulation of gene expression and protein synthesis. Irrespective of the ORF4 region’s importance, its codon usage patterns have not been determined [32, 33]. In this regard, this investigation has been carried out to analyze the codon usage patterns of the HEV ORF4 protein genes.

The codon usage analysis has been extensively carried out for protein genes of other reading frames of HEV, i.e., ORF1, ORF2 and ORF3 [34]. Baha and colleagues has evaluated the codon usage patterns of ORFs, but the codon compositional restrain in ORF4 has not been analyzed [34]. In this study, we performed comprehensive analysis of nucleotide composition and synonymous codon usage, based on available nucleotide sequences (on the NCBI GenBank) of the ORF4 protein genes, to determine the evolutionary factors that could play an important role in shaping the codon usage patterns. To the best of our knowledge, our comprehensive analysis for the first time provides insights into the codon usage patterns of ORF4 protein genes. This study will also shed lights on the distinguishing genetic features of HEV prevalent in the ORF4 sequences.

## Methods

### Sequence data acquisition

Nucleotide sequences of the ORF4 protein genes were retrieved from GenBank database available at the National Centre for Biotechnology information (NCBI) (http://www.ncbi.nlm.nih.gov). The retrieved sequences were selected based on the following inclusion criteria: (A) Selected sequences from same or different countries at varying time intervals were assembled in order to avoid repetition. (B) Sequences were included from different hosts encompassing human, rat and ferret. (C) Accumulated sequences from GenBank were categorized into different datasets. (D) Three datasets were prepared for each host organism (human, rat and ferret). (E) Multiple alignment was carried out for these datasets using ClustalW algorithm installed in the BioEdit Sequence Alignment Editor 7.2.5 [35]. The complete list of the sequences used for the present analysis in different host organisms are listed in additional files (Additional files 1–3: Tables S1–S3).

### Nucleotide composition analysis

The following nucleotide composition properties of the ORF4 sequences were calculated using Mega-X (Version 10.1.7): (1) occurrence of overall nucleotide frequencies (A%, C%, T/U% and G%); (2) occurrence of nucleotides at the third codon site (A3%, C3% U3% and G3%); and (3) occurrence of G + C content at different codon positions, i.e., first (GC1), second (GC2) and third synonymous codon positions (GC3). The five non-biased codons were omitted from the nucleotide composition analysis. It included three termination codons (UAG, UGA, UAA), i.e., as they do not code for any amino acid; and two codons AUG and UGG, as they code for particular amino acid Met and Trp, respectively, Therefore, these mentioned five codons do not exhibit any codon bias.

### Relative synonymous codon usage (RSCU) analysis

The ratio between the observed and expected usage frequency of a codon is described as the Relative Synonymous Codon Usage (RSCU). RSCU value if all synonymous codons are used equally for any specific amino acid [36]. The RSCU index was determined as follows:

$${\text{RSCU}} = \frac{{G_{ij} }}{{\sum\nolimits_{j}^{ni} {G_{ij} } }}ni$$

where *RSCU* is the relative synonymous codon usage value and *G*_*ij*_ is the observed number of the *i*th codon for the s*j*th amino acid that has an “*ni*” type of synonymous codon. Codons with RSCU values (> 1.6) and (< 0.6) were considered as “overrepresented” and “underrepresented” codons, respectively, whereas codons having the RSCU values (1) were regarded as not biased (average level codon) [37]. The mean RSCU values of the ORF4 protein genes were calculated using Mega-X (Version 10.1.7), in order to reveal the codon usage patterns without the effect of amino acid composition and sequence length.

### Relationship between overall nucleotide composition and nucleotide composition at the 3rd codon position

The correlation between A, T, G, C, GC and 3rd codon position of its counterparts (A3, T3, G3, C3, GC3) was assessed to analyze whether natural selection/mutation pressure individually contributed or both collaboratively influenced the evolution of ORF4 in HEV natural hosts.

## Results

### Analysis of nucleotide composition in coding sequences

**Fig. 1:**
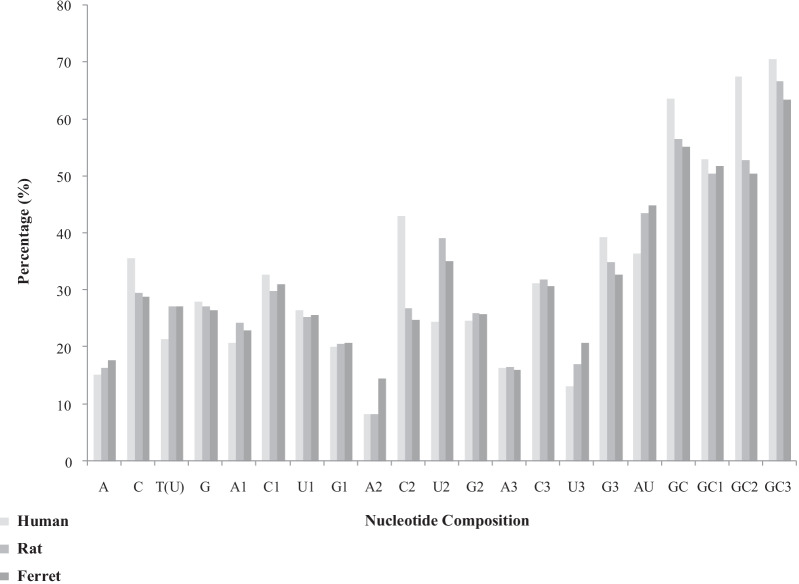
Comparative analysis of nucleotide composition patterns between HEV host organisms (human, rat and ferret)

The nucleotide compositions of the ORF4 protein genes were calculated to analyze the effect imposed by compositional constraints on codon usage. The results of the nucleotide composition analysis are mentioned in Table 1 (Fig. 1).

**Table 1:** Nucleotide composition analysis of ORF4 of hepatitis E viruses

Nucleotide	Human	Rat	Ferret
A	15.094	16.356	17.753
C	35.597	29.451	28.768
T(U)	21.341	27.122	27.119
G	27.966	27.070	26.358
A1	20.754	24.301	22.826
C1	32.704	29.891	30.978
U1	26.415	25.310	25.543
G1	20.125	20.496	20.652
A2	8.176	8.307	14.456
C2	42.893	26.708	24.728
U2	24.402	39.052	35.108
G2	24.528	25.931	25.706
A3	16.352	16.459	15.978
C3	31.194	31.754	30.597
U3	13.207	17.003	20.706
G3	39.245	34.782	32.717
AU	36.441	43.478	44.872
GC	63.563	56.498	55.126
GC1	52.829	50.387	51.63
GC2	67.421	52.639	50.434
GC3	70.439	66.536	63.314

The values are represented as percentage

*Human*: The nucleotides C and G were found to be most abundant in these coding sequences, with an average of 35.597% and 27.966%, respectively, compared with U (21.341%) and A (15.094%). The most frequent nucleotide at the third position was G3S (39.245%), followed by C3S (31.194%), A3S (16.352%) and U3S (13.207%). Thus, synonymous codons at the third position followed the same trend (G3S > C3S > A3S > U3S). The overall GC content was higher than that of AU, with 63.563% observed, compared with 36.441%, respectively, which indicated a GC-biased composition. The overall GC content and GC% at different positions GC1, GC2 and GC3 were with an average of 63.563%, 52.829%, 67.421& and 70.439%, respectively (Additional file 1: Table S1) (Table 1).

*Rat*: The nucleotides C and U were found to be most abundant in these coding sequences, with an average of 29.451% and 27.122%, respectively, compared with G (27.070%) and A (16.356%). The most frequent nucleotide at the third position was G3S (34.782%), followed by C3S (31.754%), U3S (17.003%) and A3S (16.459%). Thus, synonymous codons at the third position followed the trend (G3S > C3S > U3S > A3S). The overall GC content was higher than that of AU, with 56.498% observed, compared with 43.478%, respectively, which indicated a GC-biased composition. The overall GC content and GC% at different position GC1, GC2 and GC3 were with an average of 56.498%, 50.387%, 52.639% and 66.536%, respectively (Additional file 2: Table S2) (Table 1).

*Ferret*: The nucleotides C and U were found to be most abundant in these coding sequences, with an average of 28.768% and 27.119%, respectively, compared with G (26.358%) and A (17.753%). The most frequent nucleotide at the third position was G3S (32.717%), followed by C3S (30.597%), U3S (20.706%) and A3S (15.978%), Thus, synonymous codons at the third position followed the trend (G3S > C3S > U3S > A3S). The overall GC content was higher than that of AU, with 55.126% observed, compared with 44.872%, respectively, which indicated a GC-biased composition. The overall GC content and GC% at different position GC1, GC2 and GC3 were with an average of 55.126%, 51.63%, 50.434% and 63.314%, respectively (Additional file 3: Table S3) (Table 1).

Therefore, initially it could be interpreted that nucleotide C was overrepresented, whereas the nucleotide A was underrepresented in HEV ORF4 protein genes. The nucleotides G and T (U) were distributed randomly. In addition to this, it was observed that the GC content (> 50%) was significantly higher than AU content (since AT content was < 50%) in ORF4 protein genes.

### Analysis of codon usage patterns in coding sequences

**Fig. 2:**
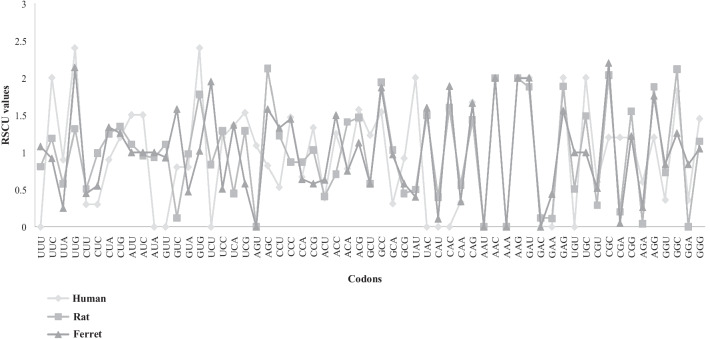
Comparative analysis of relative synonymous codon usage (RSCU) patterns between HEV- hosts (human, rat and ferret)

RSCU measure was undertaken to evaluate the codon usage pattern of ORF4 protein gene sequences. The RSCU values were computed for every codon in each gene sequence to decrypt the extent to which C-ended codons were preferred. The results are mentioned in Table 2 (Fig. 2).

**Table 2:** Average RSCU values in ORF4 of hepatitis E viruses

Amino acid	Codon	Hosts		
		Human	Rat	Ferret
Phe (F)	UUU	0	**0.81**	**1.08**
	UUC	2	**1.19**	**0.92**
Leu (L)	UUA	0.9	**0.58**	0.25
	UUG	2.4	**1.32**	**2.14**
	CUU	0.3	0.51	0.45
	CUC	0.3	**0.99**	0.55
	CUA	0.9	**1.25**	**1.34**
	CUG	1.2	**1.35**	**1.26**
Ile (I)	AUU	1.5	**1.11**	**1**
	AUC	1.5	**0.96**	**1**
	AUA	0	**0.93**	**1**
Val (V)	GUU	0	1.11	0.93
	GUC	0.8	0.12	**1.58**
	GUA	0.8	0.98	0.47
	GUG	2.4	**1.78**	1.02
Ser (S)	UCU	0	0.84	**1.95**
	UCC	**1.2**	**1.29**	0.51
	UCA	**1.36**	0.45	**1.37**
	UCG	**1.53**	**1.29**	0.58
	AGU	**1.09**	0	0
	AGC	**0.82**	**2.13**	**1.58**
Pro (P)	CCU	**0.53**	**1.23**	**1.33**
	CCC	**1.47**	0.87	**1.45**
	CCA	**0.67**	0.87	0.64
	CCG	**1.33**	**1.03**	0.58
Thr (T)	ACU	0.39	0.41	0.63
	ACC	**1.25**	0.71	1.5
	ACA	0.78	**1.41**	0.75
	ACG	**1.57**	**1.47**	1.13
Ala (A)	GCU	**1.23**	0.58	0.58
	GCC	**1.54**	**1.94**	**1.87**
	GCA	0.31	**1.03**	0.97
	GCG	**0.92**	0.45	0.58
Tyr (Y)	UAU	2	0.5	0.4
	UAC	0	1.5	**1.6**
His (H)	CAU	0	0.4	0.11
	CAC	0	1.6	**1.89**
Gln (Q)	CAA	0.33	0.56	0.34
	CAG	**1.67**	1.44	**1.66**
Arn (N)	AAU	0	0	0
	AAC	2	2	2
Lys (K)	AAA	0	0	0
	AAG	2	2	2
Asp (D)	GAU	2	1.88	2
	GAC	0	0.12	0
Glu (E)	GAA	0	0.11	0.44
	GAG	2	1.89	**1.56**
Cys (C)	UGU	0	0.51	1
	UGC	**2**	**1.49**	1
Arg (R)	CGU	0.6	0.29	0.52
	CGC	1.2	**2.04**	**2.2**
	CGA	1.2	0.2	0.05
	CGG	1.2	**1.55**	**1.22**
	AGA	0.6	0.04	0.26
	AGG	1.2	**1.88**	**1.76**
Gly (G)	GGU	0.36	0.73	0.84
	GGC	**1.82**	**2.12**	1.26
	GGA	0.36	0	0.84
	GGG	**1.45**	1.15	1.05

The preferred codons are indicated in bold

*Human*: Out of 18 preferred codons (UCC, UCA, UCG, AGU, AGC, CCU, CCC, CCA, CCG, ACC, ACG, GCU, GCC, GCG, CAG, UGC, GGC and GGG), 13 were C/G-ending (C-ending: 7; G-ending: 6) and 5 were U/A -ending (U-ending: 3; A-ending: 2) (Additional file 4: Table S4) (Table 2). This indicated preference of C-and G-ended codons over U and A-ended codons in gene sequences. Among these preferred ones, 3 had RSCU value > 1.6, i.e., overrepresented codons (CAG, UGC and GGC), while the remaining 14 had RSCU values > 0.6 and < 1.6 (UCC, UCA, UCG, AGU, AGC, CCU, CCC, CCA, CCG, ACC, ACG, GCU, GCC, GCG and GGG). Presence of one underrepresented (RSCU < 0.6) synonymous codon was revealed (CCU).

*Rat*: Out of 25 preferred codons (UUU, UUC, UUA, UUG, CUC, CUA, CUG, AUU, AUC, AUA, GUG, UCC, UCG, AGC, CCU, CCG, ACA, ACG, GCC, GCA, UGC, CGC, CGG, AGG and GGC), 17 preferred codons were C/G-ending (C-ending: 9; G-ending: 8) and 8 were U/A-ending (A-ending: 5; U-ending: 3) (Additional file 5: Table S5) (Table 2). This indicated preference of C- and G-ended codons over U- and A-ended codons in gene sequences. Among these preferred ones, 6 had RSCU value > 1.6, i.e., overrepresented codons (GUG, AGC, GCC, CGC, AGG and GGC), while the remaining 18 had RSCU values > 0.6 and < 1.6 (UUU, UUC, UUA, UUG, CUC, CUA, CUG, AUU, AUC, AUA, UCC, UCG, CCU, CCG, ACA, ACG, GCA, UGC and CGG). Presence of one underrepresented (RSCU < 0.6) synonymous codon was revealed (UUA).

*Ferret*: Out of 22 preferred codons (UUU, UUC, UUG, CUA, CUG, AUU, AUC, AUA, GUC, UCU, UCA, AGC, CCU, CCC, GCC, UAC, CAC, CAG, GAG, CGC, CGG and AGG), 15 preferred codons were C/G-ending (C-ending: 7; G-ending: 5) and 7 were U/A -ending (U-ending: 4; A-ending: 3) (Additional file 6: Table S6) (Table 2). This indicated preference of C- and G-ended codons over U and A-ended codons in gene sequences. Among these preferred ones, 7 had RSCU value > 1.6, i.e., overrepresented codons (UUG, UCU, GCC, CAC, CAG, CGC and AGG), while the remaining 15 had RSCU values > 0.6 and < 1.6 (UUU, UUC, CUA, CUG, AUU, AUC, AUA, GUC, UCA, AGC, CCU, CCC, UAC, GAG and CGG). Presence of an optional underrepresented (RSCU < 0.6) synonymous codon was not revealed.

The overall/host-specific RSCU analysis revealed that C/G-ending codons were preferred over U/A-ending codons in the ORF4 coding sequences across all host organisms. The number of preferred codons in each host followed the order: 25 (rat) > 22 (ferret) > 18 (human). Thus, our results clearly suggested the common attributes and differences among the usage of preferred codons, i.e., in the case of overrepresented and underrepresented codons in each host. Thus, our RSCU findings clearly revealed both similarities and discrepancies in the codon usage patterns among HEV-hosts.

#### Relationship among hosts by comparing codon usage frequency

A specific amino acid is encoded by more than one codon. It has been documented that the usage of synonymous codons is not random [38]. By exploiting RSCU values of the HEV-hosts, we computed the preferred codon frequency for each amino acid. The frequency was determined to analyze the influence of selection pressure from hosts on codon usage patterns of HEV. A list of preferred codons encoding amino acids with higher frequency as compared to other synonymous codons for HEV-hosts is mentioned in Table 3. (Additional files 4–6: S4–S6 Tables).

**Table 3:** Preferred codons for each amino acid in the ORF4 of HEV-hosts

Amino acid	Human	Rat	Ferret
*Phe*			
UUU(F)	0	3.9	**5.2**
UUC(F)	**3**	**5.7**	4.4
*Leu*			
UUA(L)	3	3.3	1
UUG(L)	**8**	7.4	**8.5**
CUU(L)	1	2.9	1.8
CUC(L)	1	5.6	2.2
CUA(L)	3	7	5.3
CUG(L)	4	**7.6**	5
*Iso*			
AUU(I)	**3**	**4.3**	**4.8**
AUC(I)	3	3.7	4.8
AUA(I)	0	3.6	4.8
*Val*			
GUU(V)	0	2.6	2
GUC(V)	1	0.3	**3.4**
GUA(V)	1	2.3	1
GUG(V)	**3**	**4.1**	2.2
*Ser*			
UCU(S)	0	2.4	**5.7**
UCC(S)	4.4	3.7	1.5
UCA(S)	5	1.3	4
UCG(S)	**5.6**	3.7	1.7
AGU(S)	4	0	0
AGC(S)	3	**6.1**	4.6
*Pro*			
CCU(P)	4	**4.4**	4.6
CCC(P)	**11**	3.1	**5**
CCA(P)	5	3.1	2.2
CCG(P)	10	3.7	2
*Thr*			
ACU(T)	1	1	1
ACC(T)	3.2	1.7	**2.4**
ACA(T)	2	3.4	1.2
ACG(T)	**4**	**3.6**	1.8
*Ala*			
GCU(A)	4	2	1.8
GCC(A)	**5**	**6.7**	**5.8**
GCA(A)	1	3.6	3
GCG(A)	3	1.6	1.8
*Tyr*			
UAU(Y)	**1**	1	1
UAC(Y)	0	**3**	**4**
*His*			
CAU(H)	0	0.1	0.2
CAC(H)	0	**0.6**	**3.3**
*Gln*			
CAA(Q)	1	0.7	1.7
CAG(Q)	**5**	**1.9**	**8.3**
*Asn*			
AAU(N)	0	0	0
AAC(N)	**1**	**0.9**	**0.4**
*Lys*			
AAA(K)	0	0	0
AAG(K)	**1**	**2**	**0.2**
*Asp*			
GAU(D)	**1**	**2.3**	**3**
GAC(D)	0	0.1	0
*Glu*			
GAA(E)	0	0.1	1
GAG(E)	**2**	**2.6**	**3.5**
*Cys*			
UGU(C)	0	1.7	3
UGC(C)	**7**	**5**	**3**
*Arg*			
CGU(R)	1	1	2
CGC(R)	**2**	**7.1**	**8.5**
CGA(R)	2	0.7	0.2
CGG(R)	2	5.4	4.7
AGA(R)	1	0.1	1
AGG(R)	2	6.6	6.8
*Gly*			
GGU(G)	1	1.7	2
GGC(G)	**5**	**5**	**3**
GGA(G)	1	0	2
GGG(G)	4	2.7	2.5

Comparison of codon usage frequency of preferred codons among HEV-hosts. All the preferred codons are bold indicating the highest codon frequency

The observed 10 amino acids Iso (I), Ala (A), Glu (Q), Asn (N), Lys (K), Asp (D), Glu (E), Cys (C), Arg (R) and Gly (G) showed similar usage of preferred codons, i.e., AUU for Iso, GCC for Ala, CAG for Gln, AAC for Asn, AAG for Lys, GAU for Asp, GAG for Glu, UGC for Cys, CGC for Arg and GGC for Gly, among all three natural HEV-hosts, which implicated a phenomenon of “mutual codon preference”. Therefore, the codons (AUU, GCC for Ala, CAG, AAC, AAG, GAU, GAG, CGC and GGC) indicated coincident codon usage portion, i.e., these mentioned preferred codons were commonly shared between all the natural HEV-hosts. In addition to this, within some preferred codons, discrepancies were observed between host organisms, i.e., preferred codons showed dissimilar usage among HEV-hosts For instance, HEV-hosts (human, rat and ferret) shared different usage of preferred codon for Ser (UCG for human, AGC for rat and UCU for ferret).

Moreover, this phenomenon was also observed in specific hosts, i.e., preferred codons encoding amino acid were different in specific host in comparison with other two host organisms, such as HEV-hosts (human and rat) shared evidence of preferred codon for UUC encoding Phe, except ferret, which preferred UUU over UUC; hosts human and ferret shared evidence of preferred codon for UUG encoding Leu, except rat, which preferred CUG over UUG; human and rat shared evidence of preferred codon for GUG encoding Val, except ferret, which preferred GUC over GUG; hosts human and ferret shared evidence of preferred codon for CCC encoding Pro, except rat, which preferred CCU over CCC; human and rat shared evidence of preferred codon for ACG encoding Thr, except ferret, which preferred ACC over ACG; rat and ferret shared evidence of preferred codon for UAC encoding Tyr, except human, which preferred UAU over UAC.

Our results clearly indicated that codon usage patterns in ORF4 gene sequences showed a mixture of coincidence and antagonism among HEV-hosts.

### Comparative analysis of the RSCU values among hosts

Moreover, the top most frequent used codons, least frequent used codons and unused codons also showed common attributes and differences in codon usage patterns among HEV-hosts as represented in Table 4. These observations further emphasized occurrence of mutual codon preference and lack of shared codon preference among host–pathogens.

**Table 4:** List of used codons based on frequency in ORF4 of hepatitis E viruses

Host organism	Codon (aa)	Frequency	Codon (aa)	Frequency
*Human*				
Most frequent	CCC (P)	11	GCC(A)	5
	CCG(P)	10	CCA(P)	5
	UUG(L)	8	UCA(S)	5
	UGC (C)	7	CAG(Q)	5
	UCG(S)	5.6	GGC(G)	5
Least frequent	CUU(L)	1	AAC(N)	1
	CUC(L)	1	AAG(K)	1
	GUC(V)	1	GAU(D)	1
	GUA(V)	1	CGU(R)	1
	ACU(T)	1	AGA(R)	1
	GCA(A)	1	GGU(G)	1
	UAU(Y)	1	GGA(G)	1
	CAA(Q)	1		
Not used	UUU(F)	0	AAU(N)	0
	AUA(I)	0	AAA(K)	0
	UCU(S)	0	GAC(D)	0
	UAC(Y)	0	GAA(E)	0
	CAU(H)	0	UGU(C)	0
	CAC(H)	0	AAU(N)	0
*Rat*				
Most frequent	CUG(L)	7.6	AGC(S)	6.1
	UUG(L)	7.4	UUC(F)	5.7
	CGC(R)	7.1	CUC(L)	5.6
	CUA(L)	7	CGG(R)	5.4
	GCC(A)	6.7	UGC(C)	5
	AGG(R)	6.6	GGC(G)	5
Least frequent	CAU(H)	0.1	CGA(R)	0.7
	AGA(R)	0.1	CAA(Q)	0.7
	GAC(D)	0.1	ACU(T)	1
	GAA(E)	0.1	UAU(Y)	1
	GUC(V)	0.3	CGU(R)	1
	CAC(H)	0.6		
Unused	AAA(K)	0	GGA(G)	0
	AGU(S)	0		
*Ferret*				
Most frequent	UUG(L)	8.5	UCU(S)	5.7
	CGC(R)	8.5	CUA(L)	5.3
	CAG(Q)	8.3	UUU(F)	5.2
	AGG(R)	6.8	CUG(L)	5
	GCC(A)	5.8	CCC(P)	5
Least frequent	AAG(K)	0.2	GUA(V)	1
	CAU(H)	0.2	ACU(T)	1
	CGA(R)	0.2	UAU(Y)	1
	AAC(N)	0.4	GAA(E)	1
	UUA(L)	1	AGA(R)	1
Unused codons	AAU(N)	0	GAC(D)	0
	AAA(K)	0	AGU(S)	0

The table lists codons, the amino acid (aa) encoded by the codon (in parentheses), and frequency of use (as a percentage). Codons are listed from most to least frequent. The space in the table separates the most frequently used, least frequently used and the unused codons for each host

### Effect of natural selection in shaping the codon usage patterns in HEV

It has been suggested that the frequencies of nucleotides A and U/T should be equal to that of C and G at the third position of the codon if mutational pressure affects the synonymous codon usage bias [17]. However, we observed huge variations in the nucleotide composition in the overall ORF4 gene sequences as observed in Table 1. This indicated that other mechanisms including natural selection influenced the codon usage bias in HEV. Thus, these findings concluded that compositional constraints under mutational bias in combination with natural selection shaped up the codon usage patterns in ORF4 coding sequences across all hosts.

## Discussion

As HEV exhibits enormously high genetic diversity in addition to lack of appropriate culture system for its propagation, these factors pose a major challenge in the improvement of treatment methods. HEV has been identified with multiple genotypes and subtypes via nucleotide sequence analysis [39, 40]. Characterizing genetic properties to figure out common regions and possible differences between genotypes is expected to assist and contribute to the process of a development of effective preventive measures against HEV infection. Our previous investigations have elucidated the ORF4 protein structure in different host organisms [41] in addition to its role as a probable drug target [42]. In this context, we conducted bioinformatics study of different ORF4 sequences of HEV by analyzing its codon usage patterns in different host organisms to provide insights into common attributes and differences among usage of amino acid in virus’s structure. Using these findings, it is hoped that more efficient and precise approaches could be identified and selected for treatment protocols.

The genetic code encompasses 64 codons, separated into 20 distinguishable groups. Each individual group consists of one to six codons and encodes the same amino acid. Thus, each standard amino acid is often encoded by alternative codons belonging to the same group. These alternative codons are termed as ‘synonymous’ codons. CUB is a phenomenon wherein one codon (over its synonymous partners) is preferred [15, 43]. CUB is considered as a distinctive property and appreciably differs among genes as well as genomes [36, 44]. Investigations have reported that codon usage patterns in organisms assist in the understanding of molecular organization of genomes. Due to improvement in sequencing technologies, CUB has gained more attention as codon usage patterns in several prokaryotic and eukaryotic have been studied [45]. As viruses are obligate parasites, they require a set of proteins and enzymes to colonize the host by counteracting the host’s defense mechanism [46]. The establishment of an association between a host and viruses depends on translational accuracy [47], which is largely affected by synonymous codon usage patterns [45, 48]. Mutational bias and natural selection are the two major forces that govern the overall codon usage variation in the genomes. It is well known that mutation pressure rather than translational selection is the primary determining factor of codon bias is in human RNA viruses [49]. On combining, these forces help us in decoding the selection of preferred codons that whether it has been influenced by mutational pressure or natural selection. Thus, in the presented study, we performed an orderly survey of the evolutionary pressures (i.e., mutational bias and natural selection) across the ORF4 to gain insights into its codon usgae patterns. The codon usage pattern of the reading frames (ORFs), such as ORF1, ORF2 and ORF3 protein genes have been elucidated [34]; however, our understanding of codon patterns in ORF4 remains to be determined. This study is the first in its kind to describe the codon usage of patterns of ORF4 genome of HEV in three different host organisms (human, rat and ferret).

Nucleotide composition constraints impose an effect on the codon usage patterns, and thus we performed the nucleotide composition analysis of the HEV ORF4 protein genes. The analysis revealed an overrepresentation of C nucleotide and underrepresentation of A nucleotide in the overall nucleotide composition. This is in agreement with the previous investigation carried out by Baha and colleagues in HEV isolates encompassing different genotypes and hosts [34]. The investigation revealed C as the most-represented nucleotide, while A as the least-represented nucleotide [34]. Similarly like previous observation, our nucleotide analysis also showed the random distribution of G and T (U) nucleotides [34]. Our analysis revealed that ORF4 genes were highly endowed with GC content which is again in agreement with the previous report which suggested that all the ORF coding sequences of HEV had overall high value of GC content (exceeding 50%) [34]. Our compositional characteristics revealed C/G-rich nucleotide pattern in humans, while hosts rat and ferret were observed with C/(T)U richness. These results further substantiate our findings as ORF1 and ORF3 showed C/G-rich genome, while ORF2 showed prevalence of C/T(U) nucleotides [34]. However, the observed pattern in ORF4 is different to the pattern observed in most of the RNA viruses (HIV, hepatitis C, rubella viruses), which revealed high prevalence of A rather than C [50]. This opposite nucleotide pattern biasness could be due to adaptation of a common ancestor of modern HEV strains to their host (in terms of nucleotide composition) during the process of evolution [51]. Our observed opposite patterns to majority of RNA viruses further show consistency with earlier report on other reading frames (ORF1, ORF2 and ORF3) [34]. Thus, it is interesting to mention that our findings from initial compositional analysis show consistency with the previous report on HEV ORFs codon usage patterns [34].

Next, we examined the role of selection forces in determining the codon usage patterns of ORF4 genes. In viruses, it has been suggested that their AU or GC-rich composition show correlation with RSCU patterns, such as, AU or GC-rich genomes preferred codons ending with either A/U or G/C, respectively. This trend supports the influence of mutational pressure [49]. As ORF4 revealed that nucleotide compositional bias is in line with its RSCU patterns in the case of human, mutation pressure is found to be a major driving factor in shaping its codon usage pattern. However, in the case of hosts rat and ferret, despite these regions had higher percentage of C and U nucleotides, their RSCU pattern showed preference toward C- and G-ended codons, i.e., RSCU results were not consistent with the initial nucleotide composition. This suggested the involvement of other factors besides nucleotide composition in shaping the synonymous codon usage patterns in these two host organisms (rat and ferret). In context with this, we observed huge variations in the nucleotide composition in the overall ORF4 gene sequences, which indicated that other mechanisms including natural selection influenced the codon usage bias in HEV. Thus, it could be interpreted that both mutation and natural selection forces shaped the codon usage patterns of ORF4 coding sequences. Our findings show consistency with the previous codon usage analyses carried out in HEV that demonstrated the predominance of mutation pressure [51] and natural selection, respectively [34].

Then, we next analyzed the relationship between codon usage patterns of ORF4 in its natural hosts. The common attributes and differences among HEV-hosts were scrutinized by computing the frequency of amino acids using their RSCU values. The number of preferred codons varied among different natural hosts and maximum usage was found to be in rat and least in human. Additionally, it was revealed that the number of overrepresented and underrepresented codons in each host organism also varied. Thus, a noteworthy variation in the usage for preferred codons among HEV-hosts implied that the codon usage patterns in ORF4 in different host organisms were subjected to different selection pressures. Furthermore, we observed that the frequency of the most used and least used codons also showed similarities and differences between hosts. Thus, it was revealed that HEV ORF4 showed a mixture of two codon usage patterns: coincidence and antagonism. This is similar to previous studies carried out in other viruses, such as HCV [52] and enterovirus [53]. A recent investigation on HEV has also shown both similarities and discrepancies in the ORF1 Y-domain region codon usage patterns which further substantiate our present findings [54]. It has been proposed that codon usage similar portions assist in effective translation of the corresponding amino acids between viruses and their respective hosts [55, 56], whereas the antagonistic portions of codon usage encourage in correct folding of viral proteins, even though decrease in the corresponding amino acids translation efficiency is observed [57–59]. On summing up these criteria, our findings revealed that none of the hosts showed complete resemblance or complete discrepancy to the other HEV-host.

The findings from such bioinformatics codon usage studies can be validated using experiments and further could be utilized for clinical trials to envisage our understanding of HEV biology. Such type of investigations on other viruses can shed some new lights in its behavioral biology.

## Conclusion

The presented study documents the codon usage analysis in HEV ORF4 for the first time. This novel bioinformatics approach is expected to strengthen our understanding on the common attributes and differences in the codon usage patterns among ORF4 protein genes. The nucleotide compositional analysis showed overrepresentation of C nucleotide while revealed A as the least-represented nucleotide. The synonymous codon usage analysis revealed that the preferred codons mostly ended with C and G nucleotides. Moreover, it was observed that codon usage pattern among HEV-hosts was a mixture of coincidence and antagonism. The study reveals that synonymous codon usage in ORF4 is an evolutionary process, perhaps reflecting a dynamic process of mutation and selection forces to adjust its codon usage to different hosts and conditions. Investigation of the codon usage patterns is essential for evolution and efficient expression of viral proteins so that they generate efficient immune response. Such strategies of codon optimization for preferred codon usage are very useful in vaccine development. The presented study here is anticipated to increase our knowledge regarding the mechanisms influencing codon usage and evolution of ORF4.

## Supplementary Information

**Additional file 1: Table S1**. Nucleotide composition analysis of HEV host Human in ORF4 coding sequences. 

**Additional file 2: Table S2**. Nucleotide composition analysis of HEV host Rat in ORF4 coding sequences.

**Additional file 3: Table S3**. Nucleotide composition analysis of HEV host Ferret in ORF4 coding sequences. 

**Additional file 4: Table S4**. RSCU values of the HEV host Human in ORF4 coding sequences. 

**Additional file 5: Table S5**. RSCU values of the host Rat in ORF4 coding sequences.

**Additional file 6: Table S6**. RSCU values of the HEV host Ferret in ORF4 coding sequences.

## Data Availability

Not applicable.

## Abbreviations

HEV: Hepatitis E virus
ORF4: Open reading frame 4
RSCU: Relative synonymous codon usage
